# L-Asparaginase Toxicity in the Treatment of Children and Adolescents with Acute Lymphoblastic Leukemia

**DOI:** 10.3390/jcm10194419

**Published:** 2021-09-26

**Authors:** Madalina-Petronela Schmidt, Anca-Viorica Ivanov, Daniel Coriu, Ingrith-Crenguta Miron

**Affiliations:** 1 Department Hemato-Oncology, “Sf. Maria” Children’s Hospital, 700309 Iasi, Romania; anca_vi@yahoo.com (A.-V.I.); ingridmiron@hotmail.com (I.-C.M.); 2 Department Mother and Child Care, “Grigore T Popa” University of Medicine and Pharmacy, 700115 Iasi, Romania; 3 Department Hematology, “Carol Davila” University of Medicine and Pharmacy, 020022 Bucharest, Romania; daniel_coriu@yahoo.com; 4 Department Hematology, Fundeni Clinical Institute, 022328 Bucharest, Romania

**Keywords:** asparaginase, acute lymphoblastic leukemia, toxicity, children, adolescents

## Abstract

Asparaginase is a basic component of chemotherapy in pediatric acute lymphoblastic leukemia (ALL) and has played a crucial role in improving the long-term survival of this disease. The objectives of this retrospective study were to elucidate the toxicity profile associated with asparaginase in children and adolescents with ALL, to analyze the impact of each type of toxicity on long-term outcomes, and to identify risk factors. We analyzed the medical charts of 165 patients diagnosed with ALL at Sf. Maria Iasi Children’s Hospital from 2010 to 2019 and treated according to a chemotherapeutic protocol containing asparaginase. The median duration of follow-up was 5 years (0.1–11.5 years). Groups of patients with specific types of toxicity were compared to groups of patients without toxicity. We found the following incidence of asparaginase-associated toxicity: 24.1% clinical hypersensitivity, 19.4% hepatotoxicity, 6.7% hypertriglyceridemia, 4.2% hyperglycemia, 3.7% osteonecrosis, 3% pancreatitis, 2.4% thrombosis, and 1.2% cerebral thrombosis. Overall, 82 patients (49.7%) had at least one type of toxicity related to asparaginase. No type of toxicity had a significant impact on overall survival or event-free survival. Being older than 14 years was associated with a higher risk of osteonecrosis (*p* = 0.015) and hypertriglyceridemia (*p* = 0.043) and a lower risk of clinical hypersensitivity (*p* = 0.04). Asparaginase-related toxicity is common and has a varied profile, and its early detection is important for realizing efficient and appropriate management.

## 1. Introduction

The basic component L-asparaginase is essential in the polychemotherapy protocol for acute lymphoblastic leukemia (ALL) in children, as it plays a decisive role in increasing the survival rate; as such, it is emerging as a real cornerstone of therapy [1,2]. There are published data showing that the incomplete administration of L-asparaginase, due to its significant toxicity, is associated with unfavorable outcomes in pediatric patients with ALL [3,4,5].

The mechanism of action of L-asparaginase mainly involves reducing the concentration of asparagine in both the plasma and cerebrospinal fluid (CSF), thereby depriving tumor cells of basic nutrients for protein synthesis [6].

Most normal cells can synthesize asparagine and are therefore less susceptible to the action of L-asparaginase. Tumor cells, on the other hand, require an external source of asparagine (from the plasma) due to their limited capacity to synthesize it. In particular, lymphoblasts require large amounts of asparagine to support their proliferation but, at the same time, have low levels of asparagine synthetase [6]. This explains the outstanding efficacy of L-asparaginase in therapy for acute lymphoblastic leukemia (ALL).

L-asparaginase is available in three preparations; two preparations are native, purified from bacterial sources, and one is modified from a native preparation [7]. The native preparations are derived from *E. coli* or *Erwinia chrysanthemi*. The third preparation, PEG-asparaginase, is also derived from *E. coli* and is covalently conjugated to monomethoxypolyethylene glycol, which improves the pharmacokinetics of asparaginase. The PEGylated form is better tolerated [8].

The administration of L-asparaginase is associated with an important and unique toxicity profile compared to other types of chemotherapy. Among the most important types of toxicity are allergic reactions, hepatotoxicity, hyperglycemia, diabetes, pancreatitis, thrombosis, encephalopathy, and hypertriglyceridemia [1].

Older age is considered, in some studies, to be associated with an increased risk of asparaginase-related toxicity [9]. In recent years, the treatment of ALL in the adolescent and young adult (AYA) age group has been intensively studied, for which chemotherapy protocols involving a high-intensity asparaginase regimen have been shown to lead to better survival [10,11]. Some studies have found a higher risk of pancreatitis and thromboembolism, but not hypersensitivity, in adolescents than in children <10 years [12], while other studies have reported similar rates of asparaginase-associated toxicity in AYA patients and those <16 years of age [13,14].

This study aimed to analyze the toxicity profile associated with the administration of native *E. coli*-derived L-asparaginase during the first-line chemotherapy protocol used in children and adolescents diagnosed with acute lymphoblastic leukemia and to compare the overall survival (OS) and event-free survival (EFS) of patients who showed toxicity vs. patients without toxicity. Severe toxicity can lead to the delayed administration or even discontinuation of L-asparaginase; thus, we considered it important to assess the impact of each type of toxicity on the survival and relapse rates in ALL. Additionally, as secondary objectives, we aimed to determine whether there were risk factors that predisposed patients to a specific adverse reaction to asparaginase and to compare the differences in toxicity associated with L-asparaginase between age groups, with a focus on children >10 years and adolescents >14 years.

Few studies analyzing the toxicity profile of L-asparaginase have been conducted in developing countries. We believe that it is useful to describe and analyze our experience based on the reality of a developing country, which may be helpful for those who wish to overcome the same types of problems as well as for new directions of development.

## 2. Materials and Methods

### 2.1. Patients and Treatment

We performed a retrospective observational study that included 165 patients diagnosed with acute lymphoblastic leukemia at Sf. Maria Children’s Hospital Iasi, Romania, between January 2010 and December 2019, who were treated according to the chemotherapy protocol ALL IC BFM 2002 [15] and received at least one dose of L-asparaginase.

The inclusion criteria were a diagnosis of ALL according to the WHO criteria [16], age 1–18 years, treatment according to the ALL IC BFM 2002 protocol, and the administration of at least one dose of L-asparaginase. A total of 185 patients were evaluated for eligibility, but 20 were excluded either because they did not receive at least one dose of L-asparaginase (15 patients, 3 of them due to early death and 12 who were transferred to other centers for chemotherapy) or because follow-up examinations could not be performed (5 patients). The last follow-up was performed in May 2021. The median duration of follow-up for the entire cohort of patients was 5 years (0.1–11.5 years).

The chemotherapy protocol used, ALL IC BFM 2002 [15], provides 8 doses of L-asparaginase at 5000 IU/m^2^ on Days 12, 15, 18, 21, 24, 27, 30, and 33 during the induction phase. For patients in the high-risk therapy arm, the blocks of chemotherapy during the consolidation phase involved the administration of L-asparaginase at 25,000 IU/m^2^ on Days 6 and 11 for each of the 3 high-risk chemotherapy courses. The delayed intensification protocol used 4 rounds of L-asparaginase administration at 10,000 IU/m^2^ on Days 1, 4, 8, and 11 for Protocol III of reinduction and 4 rounds of L-asparaginase at 10,000 IU/m^2^ on Days 8, 11, 15, and 18 for Protocol II of reinduction. All the L-asparaginase applications were with a native *E. coli*-derived preparation, were performed intramuscularly, and followed premedication with corticosteroids.

The response to treatment was assessed according to the criteria of the International Working Group [17]. The response on Day 8 of treatment was the response after 7 days of corticosteroid therapy and was defined as a prednisone poor response (PPR) if there were more than 1 × 10^9^/L blasts in the peripheral blood. Complete remission was defined as <5% blasts in bone marrow aspirate smears; normal erythropoiesis, granulopoiesis, and megakaryocytopoiesis; an absolute neutrophil count >1 × 10^9^/L; platelets >100 × 10^9^/L; and the absence of blasts in the cerebrospinal fluid or elsewhere. Since 2017, the response to treatment has also been evaluated in our center based on the detection of minimal residual disease (MRD) by flow cytometry [18]. Immunophenotyping was carried out using a FACS Canto II Flow Cytometer (BD Biosciences, San Jose, CA, USA).

The retrospective study of medical charts was approved by the Ethics Committees of Sf. Maria Children’s Hospital Iasi (6877/26 February 2020). Informed consent was obtained from the patients’ guardians.

### 2.2. Variables

The variables extracted and analyzed from the medical charts were the demographic characteristics of all of the patients, the presence of associated diseases, clinical features at onset (hepatomegaly, splenomegaly, adenomegaly, and hemorrhagic manifestations), CNS infiltration at onset, hematological values at onset (the white blood cell count, the platelet count, the Hb value, and the presence of blasts in the peripheral blood), the type of immunophenotypic leukemia, molecular biological abnormalities, the responses to treatment on Days 8 and 33, minimal residual disease (MRD), the type and grade of toxicity associated with asparaginase therapy (clinical hypersensitivity, hepatotoxicity, thrombosis, pancreatitis, hyperglycemia, and hypoproteinemia), relapse rates, the event-free survival probability, and the overall survival. The first author checked the coding of the categorical variables and the values of the continuous variables.

Adverse reactions and their severity were defined using version 5.0 of the Common Toxicity Criteria for Adverse Events (CTCAE) [19]. Acute pancreatitis was diagnosed according to the Atlanta criteria [20] and 2012 revised criteria [21], requiring the presence of at least 2 of the following: (1) clinical manifestations suggesting pancreatitis, (2) serum amylase or serum lipase with values 3 times higher than the upper normal limits, or (3) imaging findings suggestive of acute pancreatitis. Symptoms lasting at least 3 days were classified as a severe form of pancreatitis.

### 2.3. Genetic Analysis

Reverse-transcription polymerase chain reaction (RT-PCR) was used to detect the following fusion genes: BCR-ABL1, ETV6/RUNX1 (TEL-AML1), MLL-AF4, E2A-PBX, and SIL-TAL. These fusion genes are part of the usual molecular diagnostic panel for patients with acute lymphoblastic leukemia diagnosed at our center. The molecular biology was assessed at diagnosis, on Day 33 of treatment, on Day 1 of consolidation, and on Day 1 of the delayed intensification protocol.

### 2.4. Statistical Analysis

Statistical analysis was performed using SPSS, version 25.0 (Armonk, NY, USA). A descriptive analysis was performed using percentages and frequencies for categorical variables and medians, maxima, and minima for continuous quantitative variables. Bivariate analysis was used to analyze the relationships and correlations between the observed variables. We used multiple logistic regression analysis to assess the association between 2 or more independent variables and a single dependent variable. Variables with *p*-values < 0.2 at the bivariate level were considered in the multivariate analysis for each type of toxicity. Age and gender variables were included in each model. The Kaplan–Meier method was used to estimate the overall survival (OS) and event-free survival (EFS), and the subgroups were compared using log-rank tests. OS is defined as the time from diagnosis to death from any cause or the date of the last follow-up. The EFS was calculated as the time interval from the date of diagnosis to the date of the first event, consisting of recurrence, resistance, death, or second malignancy, or the date of the last follow-up if no event occurred. *p*-values were derived from 2-sided tests and were considered significant if <0.05. The Cox proportional-hazards regression model was used for univariate and multivariate survival analysis.

## 3. Results

Among the 165 patients included in our study, 82 (49.7%) had at least one type of toxicity related to asparaginase.

Table 1 comparatively summarizes the incidence of different types of asparaginase-associated toxicity for all the patients by age group (≥10 years vs. <10 years).

We noted that patients aged >10 years had more frequent hyperglycemia (*p* = 0.001) and osteonecrosis (*p* = 0.003). The median age at diagnosis for patients with severe hyperglycemia was 13.4 years (4.9–17.1 years), and 85.7% of them were ≥10 years old at diagnosis (*p* = 0.001; odds ratio: 16.047; 95% CI: 1.877–137.179).

Regarding the group of adolescents >14 years of age included in our study, univariate logistic regression analysis showed that they had a higher risk of osteonecrosis (*p* = 0.015) and hypertriglyceridemia (*p* = 0.043) and a lower risk of clinical hypersensitivity (*p* = 0.04) (Table 2).

### 3.1. L-Asparaginase Clinical Hypersensitivity

Clinical hypersensitivity was the most common type of toxicity encountered in our sample, occurring in 40 patients (24.1%). We note that a premedication protocol (hydrocortisone or antihistamine drugs) was used before each dose of asparaginase, even if the patient had not previously had an allergic reaction.

In terms of severity, only five patients (12.5%) had allergic reactions of grade ≥3 according to CTCAE. There were no deaths due to allergic reactions. The EFS rate in patients with CTCAE grade ≥3 hypersensitivity was 60% compared to 80% in the patients with grade <3 hypersensitivity (*p* = 0.238), and the OS rates were 80 and 82.9%, respectively (*p* = 0.823).

Allergic reactions occurred in the first hour after the intramuscular administration of L-asparaginase (28 patients, 70%), but there were also 12 cases (30%) in which a local allergic reaction occurred 1–2 days after administration. The occurrence of a clinical hypersensitivity reaction was observed in six cases (15%) during the induction phase, five cases (12.5%) during the administration of high-risk blocks in the consolidation phase, and 29 cases (72.5%) during the delayed intensification phase.

In 19 of the 40 cases of clinical hypersensitivity (47.5%), L-asparaginase from *E. coli* was replaced with *Erwinia chrysanthemi*-derived asparaginase, and for 10 patients (25%), it was replaced with PEG-asparaginase; 11 patients (27.5%) discontinued asparaginase.

Patients who switched to another type of asparaginase had no associated hypersensitivity.

**Figure 1:**
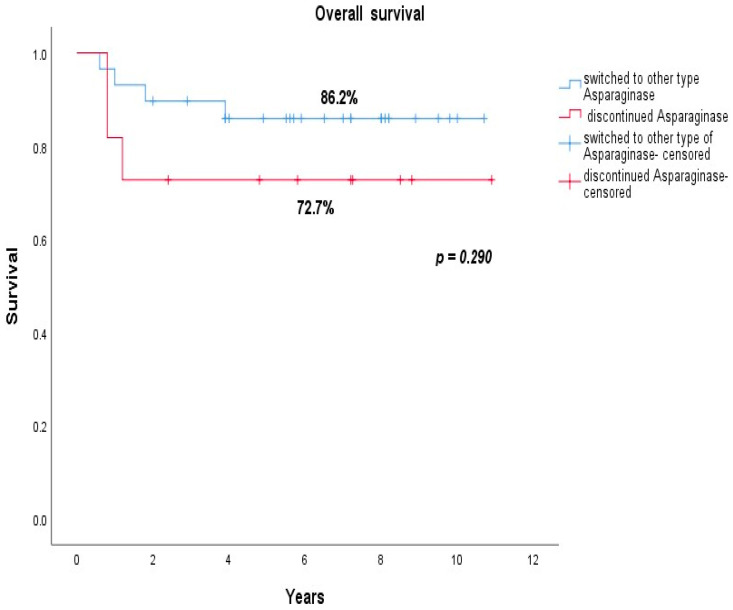
Overall survival of patients who switched to another type of asparaginase (blue line) vs. patients who discontinued asparaginase (red line).

As shown in Figure 1, the overall survival of patients who continued the therapy with another type of asparaginase product was 86.2% versus 72.7% in the group of patients who discontinued asparaginase (*p* = 0.290).

Multivariate Cox regression analysis (adjusted for age <10 years and prednisone-good-response variables, known prognostic factors for favorable outcomes) showed that replacing asparaginase with another type of asparaginase preparation was not a predictive factor for overall survival: adjusted HR: 0.394; 95% CI: 0.085–1.828; *p* = 0.234.

Table 3 shows the comparative clinical–biological features, outcomes, and survival of patients with and without clinical hypersensitivity.

**Figure 2:**
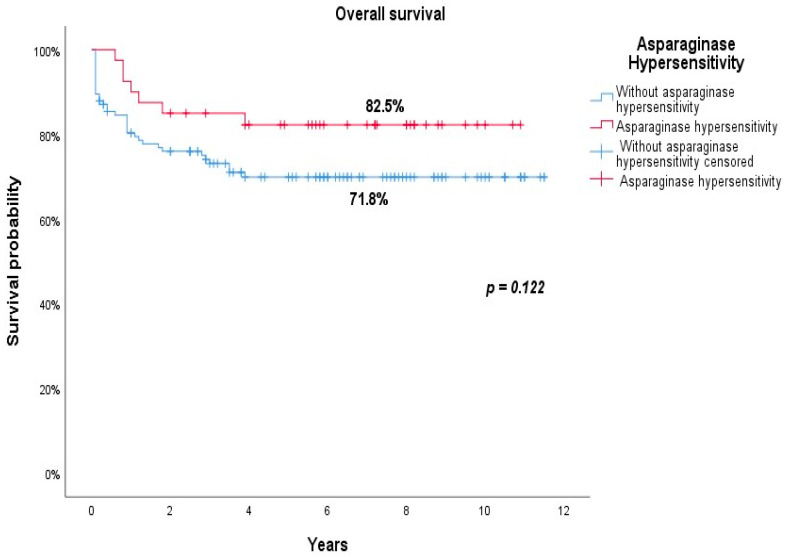
Overall survival of patients with (red line) and without (blue line) clinical hypersensitivity.

**Figure 3:**
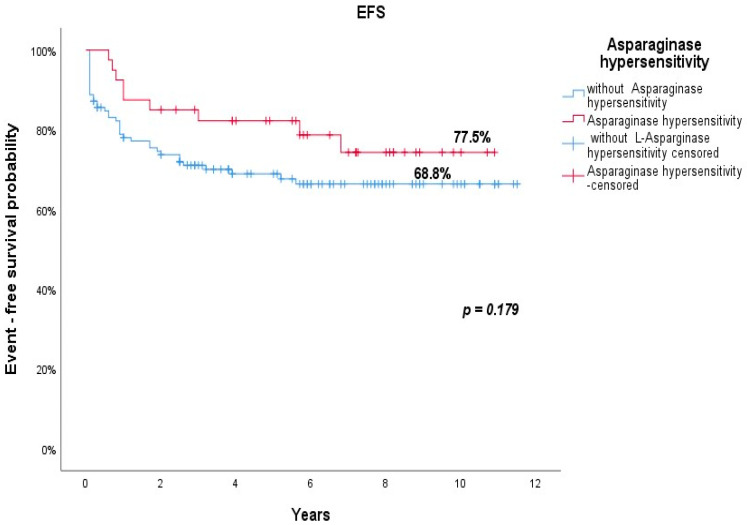
EFS of patients with (red line) and without (blue line) clinical hypersensitivity.

As shown in Figure 2, the OS was 82.5% in the group of patients who had clinical hypersensitivity to L-asparaginase, compared to 71.8% in those without an allergic reaction (82.5% vs. 71.8%; *p* = 0.122). As illustrated in Figure 3, the EFS was 77.5% for patients who showed clinical hypersensitivity to L-asparaginase versus 71.8% in those without clinical hypersensitivity (*p* = 0.179).

Multivariate Cox regression analysis adjusted for variables associated with improved overall survival (age of 1–6 years, a good prednisone response, and the WBC count at diagnosis) revealed that asparaginase hypersensitivity was not an independent predictor for OS (adjusted HR: 0.506; 95% CI: 0.223–1.147; *p* = 0.103) and EFS (adjusted HR: 0.584; 95% CI: 0.280–1.214; *p* = 0.150) (Table 4).

### 3.2. L-Asparaginase Hepatotoxicity

Hepatotoxicity was the second-most-observed side effect associated with asparaginase. We found that 32 (19.4%) of the patients in our study had L-asparaginase hepatotoxicity. In terms of severity, 20 patients (62.5%) had CTCAE grade ≥ 3 hepatotoxicity and 12 patients (37.5%) had grade < 3. Three patients (12.5%) had grade 5 hepatotoxicity with fulminant liver failure and death.

**Figure 4:**
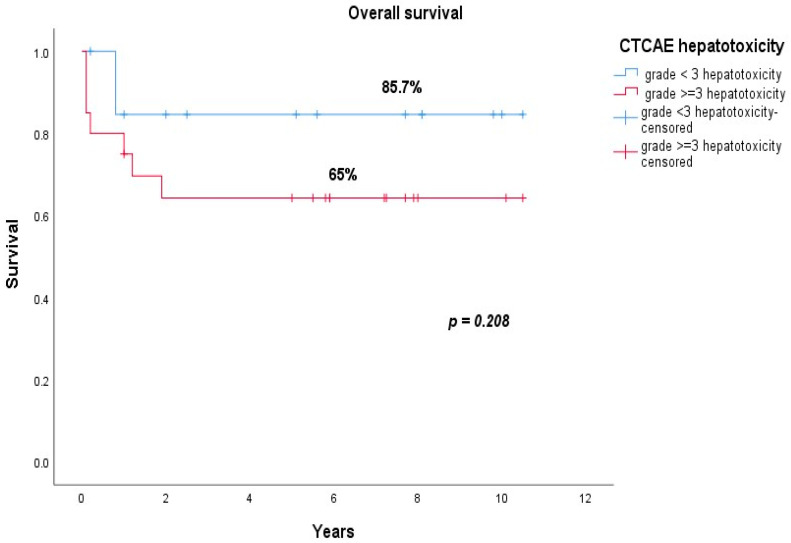
EFS of patients with CTCAE grade < 3 hepatotoxicity (blue line) vs. grade ≥ 3 hepatotoxicity (red line).

The EFS rate for patients with CTCAE grade ≥ 3 hepatotoxicity was 65%, compared to 85.7% for patients with grade < 3 hepatotoxicity (*p* = 0.208) (Figure 4), and the OS rates were 65% and 85.7%, respectively; *p* = 0.227.

The multivariate Cox regression analysis of OS and EFS of patients with CTCAE grade ≥ 3 hepatotoxicity revealed that it was not an independent predictor for worse OS (adjusted HR: 1.861; 95% CI: 0.739–4.369, *p* = 0.153) or EFS (adjusted HR: 1.520; 95% CI: 0.660–3.497, *p* = 0.325) (Table 5).

All three patients who had acute liver failure and who died had this type of toxicity during the induction protocol when corticosteroid therapy was given with L-asparaginase. We noted that none of the three patients who died had a previous history of liver disease, and all three had normal liver ultrasounds before chemotherapy.

Table 6 shows the biological features, responses to treatment, relapse rates, and overall survival (OS) and event-free survival (EFS) rates for the group of patients who presented hepatotoxicity (all grades) vs. those who did not show hepatotoxicity.

### 3.3. Thrombosis

In the cohort of patients analyzed, thrombosis was identified in four patients (2.4%); two of them had cerebral thrombosis (1.2%), and the other two had internal jugular vein thrombosis (1.2%). The two cases of cerebral thrombosis were diagnosed in the induction phase of the protocol, and the other two cases were diagnosed in the reinduction phase. There was one death due to extensive cerebral thrombosis.

It is noteworthy that, of the patients who had thrombosis, three (75%) had a positive TEL-AML1 rearrangement at the time of disease onset (*p* = 0.001; odds ratio: 3.439; 95% CI: 0.629–18.80).

### 3.4. Hyperglycemia

Seven patients (4.2%) in the study group had hyperglycemia that required insulin administration, which was grade 3 (five patients) or grade 4 (two patients) according to the CTCAE criteria. No patient died, and in all cases, the hyperglycemia was transient and remitted after L-asparaginase was discontinued. The onset of hyperglycemia occurred during the induction protocol in three patients (42.8%) and during the reinduction protocol in four patients (57.1%). One patient (0.6%) had diabetic ketoacidosis, and two patients also had pancreatitis.

Interestingly, all seven patients with severe hyperglycemia were male. One of the patients had an associated congenital cataract.

### 3.5. Pancreatitis and Hypertriglyceridemia

Of the 165 patients included in the study, five had asparaginase-associated pancreatitis (AAP) (3%), three mild and two severe. One patient had an unfavorable outcome and died.

Eleven patients (6.7%) had hypertriglyceridemia of grade ≥ 3 according to CTCAE.

Univariate logistic regression analysis showed a statistically significant correlation between hypertriglyceridemia and pancreatitis (*p* = 0.002; odds ratio: 11.185; 95% CI: 1.654–75.641).

Two of the patients with AAP had a significant family history of neoplasms; one child had a brother who died of medulloblastoma, and another child had three relatives with grade 2 malignant hematologic disorders. Another patient with APP had Down’s syndrome and ALL.

All five cases of pancreatitis were diagnosed during the delayed intensification phase of treatment. The patients who had mild forms of pancreatitis resumed asparaginase, and the pancreatitis did not recur.

Table 7 shows the biological features and outcomes of the group of patients with AAP vs. patients who did not have AAP.

**Figure 5:**
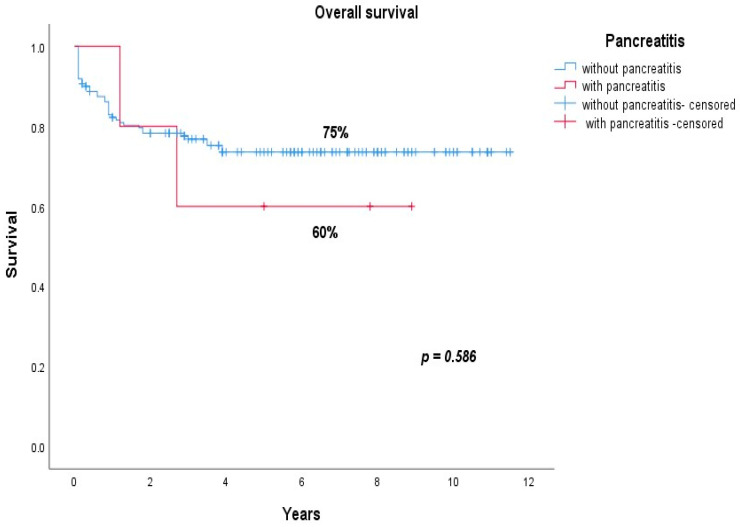
Overall survival of patients who had pancreatitis (red line) compared to those who did not (blue line).

The overall survival was 60% for patients who had pancreatitis compared to 75% for patients without pancreatitis (*p* = 0.586) (Figure 5).

### 3.6. Multivariate Cox Regression Analysis

Multivariate Cox regression analysis with adjustments for known predictors of overall survival (age < 6 years, good prednisone response, and WBC < 50,000/mm^3^ at diagnosis) was used to describe the association between each type of asparaginase-related toxicity and overall survival (Table 8). The results show that no type of toxicity was predictive of worse or better overall survival.

## 4. Discussion

Asparaginase-associated toxicity was common, 82 (49.7%) of the 165 patients included in our study developing at least one type of asparaginase-related side effect. There were five deaths (3% of patients) related to asparaginase toxicity. Asparaginase-associated adverse events that led to death were hepatotoxicity (three cases), pancreatitis (one case), and cerebral thrombosis (one case).

The protocols for the prophylactic and diagnostic procedures regarding asparaginase-related complications used in our center include the following: patients receiving premedication with hydrocortisone or antihistamine drugs before each dose of asparaginase; the twice-weekly laboratory monitoring of complete blood counts (CBCs), serum glucose, liver function, fibrinogen, and albumin; the weekly laboratory monitoring of serum amylase and triglycerides; and abdominal ultrasound if the patient is symptomatic. The monitoring of anti-asparaginase antibodies is not available in our center. Additionally, monitoring AT III levels during asparaginase therapy is not routinely performed, and no anticoagulant prophylactic therapy is administered.

We found that allergic reactions were less common for older children, and this finding is consistent with the study of Schmiegelow and Rank, who showed that the risk of L-asparaginase allergy decreased with age [12].

The incidence of hypersensitivity (24.1%) observed in our study is comparable to that found in other published studies, in which incidence rates of 10 to 30% have been described [1,22,23,24,25,26]. A study by Liu et al. analyzed patients treated according to the frontline St. Jude Total XV protocol, which uses a higher dose of asparaginase during induction (10,000 UI/m^2^ on Days 6, 8, 10, 12, 14, and 16) than that used in the ALL IC BFM 2002 protocol (5000 UI/m^2^ on Days 12, 15, 18, 21, 24, 27, 30, and 33), and reported a higher incidence of clinical allergy (41%) [27].

An “a priori” prophylactic protocol against hypersensitivity (hydrocortisone or antihistamine drugs) was used in our patients to prevent L-asparaginase discontinuation and this could mask allergic symptoms and therefore some of the patients may have reduced asparaginase activity due to the anti-asparaginase antibodies. Furthermore, premedication in patients who have already experienced a hypersensitivity reaction may not protect against a further hypersensitivity [28]. However, a recent study by McCormick et al. illustrated that the use of premedication prior to PEG-asparaginase combined with asparaginase level monitoring is the most cost-effective approach in both standard and high risk pediatric ALL patients with 8% and 7% fewer changes to *Erwinia*-derived asparaginase compared to using monitoring without premedication [29].

In our study, all 40 patients who had clinical hypersensitivity discontinued native *E. coli* L-asparaginase. Most of them (29) switched to another type (*Erwinia*-derived asparaginase or PEG-asparaginase), but 11 patients did not receive any type of asparaginase preparation after the allergic episode. Among the 11 patients who discontinued asparaginase, seven had a cumulative dose ≥50% of the scheduled dose. During the study period, *Erwinia*-derived asparaginase or PEG-asparaginase were not always available in our country. Some patients purchased PEG-asparaginase from other countries.

Some studies have shown that discontinuing asparaginase can result in a negative course for the underlying disease (ALL) [5]. We found an overall survival rate of 86.2% in the group of patients who switched to another type of asparaginase preparation, compared to 72.7% in the patients who discontinued asparaginase (*p* = 0.290), with no statistically significant difference in terms of the OS between the two groups. Multivariate Cox regression analysis (adjusted for age < 10 years and prednisone-good-response variables, known prognostic factors for favorable outcomes) showed that replacing asparaginase with another type of preparation was not a predictive factor for overall survival (adjusted HR: 0.394; 95% CI: 0.085–1.828; *p* = 0.234); however, the data should be interpreted with caution due to the small number of patients.

This finding is consistent with a study by Yen et al., who showed that patients who discontinued asparaginase due to allergic reactions and patients who continued treatment with *Erwinia*-derived asparaginase had similar 5-year OS and EFS [30].

The incidence of liver failure associated with asparaginase was only 1.8%, similar to the 1% reported in the study of Advani et al. [31] Although liver failure is rare, it led to the death of three patients, two of whom had favorable prognostic factors for the underlying disease (TEL-AML1 rearrangement and age < 6 years). We identified the induction period of treatment after six applications of asparaginase as a critical time point for severe hepatotoxicity; all three cases of liver failure were diagnosed after the sixth dose of L-Asp in the chemotherapy protocol. None of these patients had a previous history of liver disease, and all had normal liver ultrasounds before chemotherapy.

It is important to note the macrovacuolar steatosis associated with hepatocytic necrosis that was described in the necropsy examinations of two of the three patients who died of liver failure. This was also described in the study of Kamal et al. regarding a liver biopsy performed in a patient who had severe liver toxicity associated with *E. coli* L-asparaginase [32].

The OS rate for patients with CTCAE grade ≥ 3 hepatotoxicity was 65%, compared to 85.7% in the group of patients with CTCAE grade < 3 hepatotoxicity; however, multivariate Cox regression analysis revealed (after adjustments for confounding variables) that grade ≥ 3 hepatotoxicity was not an independent predictor for worse OS (adjusted HR: 1.861; 95% CI: 0.739–4.369; *p* = 0.153) or EFS (adjusted HR: 1.520; 95% CI: 0.660–3.497; *p* = 0.325). The presence of CTCAE grade ≥ 3 hepatotoxicity delayed the next dose of asparaginase until it returned to grade ≤ 2 in 15 patients. An in-depth analysis of the impact of dose delay on overall survival could not be performed, as detailed data on the number of delayed days for each dose and the cumulative duration of delay for each patient were not available.

We found, in our cohort of patients, a 2.4% incidence of thrombosis associated with asparaginase and a 1.2% incidence of cerebral thrombosis. Caruso et al. published a meta-analysis of 17 prospective studies that included 1752 pediatric patients with acute lymphoblastic leukemia, and they calculated the rate of thrombosis at 5.2%, with most thromboses being diagnosed during induction, and the total risk of cerebral thrombosis at 2.9% [33].

The lower incidence of thrombosis identified in our study can be explained by the lower percentage of patients who had central venous catheters during induction (<25%) and the fact that dexamethasone was used as corticosteroid therapy during the induction phase. There are data in the literature associating dexamethasone with a decreased risk of thrombotic events during the induction phase in the treatment of ALL in children [34].

The two cases of cerebral thrombosis, one venous and one arterial, occurred during induction and after the third and sixth doses of asparaginase, respectively.

One of the children diagnosed with cerebral thrombosis had thrombophilia (the association of a heterozygous Leiden factor V mutation, heterozygous factor II G 20210A mutation, and homozygous MTHFR C677T gene mutation) as a risk factor. Caruso et al. showed that hereditary thrombophilia increased the risk of thrombosis in pediatric patients treated with asparaginase by 8.5 times [33], which is why we consider thrombophilia screening at the beginning of treatment to be useful. Our patient continued the treatment with asparaginase after clinical and imaging improvement concomitantly with low-molecular-weight heparin (LMWH) therapy. The patient is now in complete remission 4 years and 3 months after the onset of ALL.

We did not find studies on the safety of re-exposure to asparaginase after cerebral thromboembolism. However, there is a study that analyzed prophylactic treatment with LMWH during induction treatment [35]. In our center, no anticoagulant prophylactic therapy is administered, and AT III levels are not routinely monitored during asparaginase therapy. An interesting correlation to note is that between thrombosis and TEL-AML1 rearrangement (*p* = 0.001; odds ratio: 3.439; 95% CI: 0.629–18.800); however, given the small number of patients diagnosed with thrombosis, the results should be interpreted with caution.

Asparaginase-induced hyperglycemia can be explained by the fact that asparaginase decreases insulin production and insulin-receptor expression [1,36]. In our study, 4.2% of the patients required insulin administration, and in all cases, it was transient, remitting after the end of treatment. The low percentage of patients with diabetic ketoacidosis in our study (0.6%) is similar to that found in the study of Robertson et al. (0.8%) [37]

Of the 165 patients included in the study, only 3% had asparaginase-associated pancreatitis (AAP), and there was a statistically significant correlation between hypertriglyceridemia and pancreatitis (*p* = 0.002; odds ratio: 11.185; 95% CI: 1.654–75.641).

Two of the patients who developed pancreatitis had a significant family history of neoplastic disease at a young age, and one patient had associated Down’s syndrome, suggesting a genetic predisposition that would lead to this type of toxicity. Polymorphisms in the sequences of some genes (CFTR, CTRC, PRSS1, and PRSS2) that have been associated with the risk of pancreatitis in general have been described in the literature [38], and in 2016, Liu et al. published a study reporting the finding of a nonsense variant of the CPA2 gene encoding carboxypeptidase A2 associated with an increased risk of AAP [39].

The patients in our study who had mild pancreatitis continued asparaginase therapy without the recurrence of episodes of AAP, which is consistent with the study conducted by Raja et al. [40], who showed that re-exposure to asparaginase in patients with mild pancreatitis is safe.

Although we analyzed a relatively large cohort of patients, our study has several important limitations related to its retrospective character and lack of analysis of anti-asparaginase antibody data. Additionally, we were unable to monitor the asparaginase activity and AT III levels.

In our cohort of patients, the treatment-related mortality (TRM) associated with asparaginase toxicity was 3%. However, multivariate Cox regression analysis (with adjustments for known prognostic factors for favorable outcomes) showed that the occurrence of any type of toxicity did not have a significant impact on the overall survival of pediatric ALL patients.

As asparaginase is a critical component of the ALL chemotherapy protocol, it is important to be aware of asparaginase-associated toxicity and to detect it at an early stage in order to enable efficient and appropriate management and continue therapy as much as possible.

## Figures and Tables

**Table 1:** Incidence of L-asparaginase-associated toxicity.

Type of Toxicity	Total Number of Patients *n* = 165	Age > 10 Years *n* = 49	Age < 10 Years *n* = 116	*p*-Value
Clinical hypersensitivity	40 (24.1%)	7 (4.3%)	33 (28.4%)	0.053
Hepatotoxicity	32 (19.4%)	6 (12.2%)	26 (22.4%)	0.133
Severe hypoproteinemia	15 (9.1%)	3 (6.1%)	12 (10.3%)	0.390
Hyperglycemia	7 (4.2%)	6 (12.2%)	1 (0.9%)	**0.001**
Hypertriglyceridemia	11 (6.7%)	6 (12.2%)	5 (4.31%)	0.063
Pancreatitis	5 (3%)	2 (4.1%)	3 (2.6%)	0.610
Osteonecrosis	6 (3.7%)	5 (10.2%)	1 (0.9%)	**0.003**
Thrombosis Cerebral thrombosis	4 (2.4%) 2 (1.2%)	1 (2%) 0 (0%)	3 (2.6%) 2 (1.72%)	0.836

**Table 2:** Incidence of L-asparaginase-associated toxicity in adolescents >14 years.

Type of Toxicity	Adolescents > 14 Years *n* = 25	OR 95% CI	*p*-Value
Clinical hypersensitivity	2 (8%)	0.233 0.52–0.968	**0.04**
Hepatotoxicity	2 (8%)	0.319 0.071–1.429	0.119
Severe hypoproteinemia	3 (12%)	1.455 0.380–5.575	0.584
Hyperglycemia	2 (8%)	2.348 0.430–12.831	0.314
Hypertriglyceridemia	4 (16%)	3.619 1.025–13.438	**0.043**
Pancreatitis	2 (8%)	3.971 0.629–25.076	0.117
Osteonecrosis	3 (12%)	6.227 1.181–32.830	**0.015**
Asparaginase toxicity	9 (36%)	0.516 0.214–1.246	0.138

Multivariate logistic regression analysis revealed that age >10 years was a risk factor for hyperglycemia (*p* = 0.014; odds ratio: 15.758; 95% CI: 1.738–142.875), age >14 years was a risk factor for hypertriglyceridemia (*p* = 0.038; odds ratio: 4.403; 95% CI: 1.085–17.861), and age >14 years (*p* = 0.014; odds ratio: 10.800; 95% CI: 1.630–71.557), female gender (*p* = 0.038; odds ratio: 8.517; 95% CI: 1.123–64.579), and hypertriglyceridemia (*p* = 0.049; odds ratio: 9.273; 95% CI: 1.013–84.890) were risk factors for osteonecrosis.

**Table 3:** Biological features and outcomes of patients with and without L-asparaginase clinical hypersensitivity.

	Asparaginase Hypersensitivity	Without Asparaginase Hypersensitivity	*p*-Value
Number	40 (24.1%)	125 (75.3%)	
Median age at diagnosis (years)	4.8	5.9	0.056
Sex			0.489
Male	28 (70%)	80 (64%)	
Female	12 (30%)	45 (36%)	
Median WBC count (range)	13,020/mm^3^ (970–348,000/mm^3^)	12,250 (420–1,000,000/mm^3^)	0.747
Median Hb concentration (range)	6.1 g/dL (2.9–12.8)	7.1 (2–13.5)	0.086
Median Plt count (range)	33,000/mm^3^ (2000–302,000/mm^3^)	38,500 (3000–573,000/mm^3^)	0.193
High-risk therapy	11 (27.5%)	22 (17.6%)	0.174
Prednisone poor response	7 (17.5%)	16 (12.8%)	0.468
(PPR)			
T-ALL/BCP-ALL	6/34 (15%/85%)	20/105 (16%/84%)	0.88
CNS infiltration			0.183
Yes	1 (2.5%)	11 (8.8%)	
No	39 (97.5%)	114 (91.2%)	
Molecular abnormalities			
E2A-PBX	1 (2.5%)	6 (4.8%)	0.532
TEL-AML1	6 (15%)	19 (15.2%)	0.978
MLL-AF4	2 (5%)	1 (0.8%)	0.084
Relapse	7 (17.5%)	17 (13.6%)	0.557
Bone marrow relapse	6 (15%)	13 (10.4%)	
CNS relapse	1 (2.5%)	4 (3.2%)	
Early relapse	6 (15%)	13 (10.4%)	
Late relapse	0 (0%)	4 (3.2%)	
OS	82.5%	71.8%	0.122
EFS	77.5%	68.8%	0.179

WBC, white blood cell; Hb, hemoglobin; Plt, platelet; T-ALL, T-cell acute lymphoblastic leukemia; BCP-ALL, B-cell precursor acute lymphoblastic leukemia; CNS, central nervous system; OS, overall survival; EFS, event-free survival.

**Table 4:** Multivariate Cox regression analysis of the impact of asparaginase hypersensitivity on outcome.

	*p*-Value	HR	95% CI
**OS**			
Unadjusted	0.138	0.541	0.240–1.219
Adjusted for age < 6 years, good prednisone response, and WBC < 50,000/mm^3^ at diagnosis	0.103	0.506	0.223–1.147
**EFS**			
Unadjusted	0.189	0.615	0.298–1.270
Adjusted for age < 6 years, good prednisone response, and WBC < 50,000/mm^3^ at diagnosis	0.150	0.584	0.280–1.214

**Table 5:** Multivariate Cox regression analysis of the impact of grade ≥3 hepatotoxicity on outcome.

	Hepatotoxicity Grade ≥ 3		
	*p*-Value	HR	95% CI
OS			
Unadjusted	0.357	1.467	0.649–3.317
Adjusted for age < 6 years, good prednisone response, and WBC < 50,000/mm^3^ at diagnosis	0.153	1.861	0.739–4.369
EFS			
Unadjusted	0.626	1.222	0.546–2.732
Adjusted for age < 6 years, good prednisone response, and WBC < 50,000/mm^3^ at diagnosis	0.325	1.520	0.660–3.497

**Table 6:** Biological features and outcomes of patients with and without L-asparaginase hepatotoxicity.

	Asparaginase Hepatotoxicity	Without Asparaginase Hepatotoxicity	*p*-Value
Number	32 (19.4%)	133 (80.6%)	
Median age at diagnosis (years)	4.9 (1.5–15.9)	5.9 (1–17.1)	0.192
Sex			0.422
Male	19 (59.4%)	89 (66.9%)	
Female	13 (40.6%)	44 (33.1%)	
Median WBC count (range)	14,765/mm^3^ (640–479,000)	12,140/mm^3^ (420–1,000,000)	0.861
Median Hb concentration (range)	6.2 g/dL (2–13.4)	7 g/dL (2.4–13.5)	0.867
Median Plt count (range)	33,000/mm^3^ (3000–302,000)	38,000/mm^3^ (3000–302,000)	0.853
High-risk therapy	4 (12.5%)	29 (21.8%)	0.239
Prednisone poor response	3 (9.4%)	20 (15%)	0.4
(PPR)			
T-ALL/BCP-ALL	5/27 (15.6%/84.4%)	21/112 (15.8%/84.2%)	0.982
CNS infiltration			0.316
Yes	1 (3.1%)	11 (8.3%)	
No	31 (96.9%)	122 (91.7%)	
Molecular abnormalities			
E2A-PBX	1 (3.2%)	6 (4.5%)	0.763
TEL-AML1	7 (21.9%)	18 (13.5%)	0.192
MLL-AF4	0	3 (2.3%)	0.404
Relapse	3 (9.4%)	21 (15.8%)	0.387
Bone marrow relapse	2 (6.2%)	16 (12%)	
CNS relapse	1 (3.1%)	5 (3.7%)	
Early relapse	3 (9.4%)	16 (15.8%)	
Late relapse	0 (0%)	5 (3.7%)	
OS	75%	74.2%	0.982
EFS	75%	69.9%	0.648

WBC, white blood cell; Hb, hemoglobin; Plt, platelet; T-ALL, T-cell acute lymphoblastic leukemia; BCP-ALL, B cell precursor acute lymphoblastic leukemia; CNS, central nervous system; OS, overall survival; EFS, event-free survival.

**Table 7:** Biological features and outcomes of patients with and without AAP.

	Asparaginase-Associated Pancreatitis	Without Asparaginase-Associated Pancreatitis	*p*-Value
Number	5 (3%)	160 (97%)	
Median age at diagnosis (years)	5.9 (4.9–17.1)	5.3 (1–17)	0.157
Adolescents ≥ 14 years	2 (40%)	23 (14.4%)	0.117
Sex			0.795
Male	3 (60%)	105 (65.6%)	
Female	2 (40%)	55 (34.4%)	
Median WBC count (range)	4000/mm^3^ (2900–16,840)	13,500/mm^3^ (420–1,000,000)	0.35
Median Hb concentration (range)	11.5 g/dL (5.9–13.4)	6.7 g/dL (2–13.5)	0.004
Median Plt count (range)	144,000/mm^3^ (23,000–246,000)	36,000/mm^3^ (2000–573,000)	0.08
High-risk therapy	1 (20%)	32 (20%)	
Prednisone poor response	0 (0%)	23 (14.4%)	0.361
(PPR)			
T-ALL/BCP-ALL	1/4 (20%/80%)	25/135 (15.6%/84.4%)	0.792
CNS infiltration			0.526
Yes	0 (0%)	12 (7.5%)	
No	5 (100%)	148 (92.5%)	
Molecular abnormalities			
E2A-PBX	0 (0%)	7 (4.4%)	0.629
TEL-AML1	0 (0%)	25 (15.6%)	0.332
MLL-AF4	0 (0%)	3 (1.9%)	0.755
Relapse	1 (20%)	23 (14.4%)	0.731
OS	60%	75%	0.586
EFS	60%	71.3%	0.727

WBC, white blood cell; Hb, hemoglobin; Plt, platelet; T-ALL, T-cell acute lymphoblastic leukemia; BCP-ALL, B cell precursor acute lymphoblastic leukemia; CNS, central nervous system; OS, overall survival; EFS, event-free survival.

**Table 8:** Multivariate Cox regression analysis of patients with asparaginase toxicity dataset.

Overall Survival			
	*p*-Value *	HR *	95%CI *
**Grade < 3 hepatotoxicity**	0.665	0.727	0.172–3.075
**Grade ≥ 3 hepatotoxicity**	0.153	1.861	0.793–4.369
**Hypersensitivity**	0.103	0.506	0.223–1.147
**Pancreatitis**	0.311	2.115	0.496–9.013
**Thrombosis**	0.079	3.685	0.860–15.793
**Asparaginase toxicity**	0.346	0.741	0.397–1.383

* Adjusted values.

## Data Availability

The data are available on request due to privacy restrictions.
